# Circulating miR-320a-3p and miR-483-5p level associated with pharmacokinetic–pharmacodynamic profiles of rivaroxaban

**DOI:** 10.1186/s40246-022-00445-5

**Published:** 2022-12-28

**Authors:** Hanxu Zhang, Zhuo Zhang, Zhiyan Liu, Guangyan Mu, Qiufen Xie, Shuang Zhou, Zhe Wang, Yu Cao, Yunlong Tan, Xiaohua Wei, Dongdong Yuan, Qian Xiang, Yimin Cui

**Affiliations:** 1 Department of Pharmacy, Peking University First Hospital, No. 8, Xishiku Street, Xicheng District, Beijing, 100034 China; 2 School of Pharmaceutical Sciences, Peking University Health Science Center, Beijing, China; 3 Office of Drug Clinical Trial Management, Affiliated Hospital of Qingdao University, Qingdao, Shandong China; 4 Psychiatry Research Center, Beijing HuiLongGuan Hospital, Peking University, Beijing, China; 5 Clinical Trial Research Center, Department of Pharmacy, The First Affiliated Hospital of Nanchang University, Nanchang, Jiangxi China; 6 Department of Pharmacy, The 7Th People’s Hospital of Zhengzhou, Zhengzhou, Henan China; 7 Institute of Clinical Pharmacology, Peking University, Beijing, China

**Keywords:** Circulating microRNA, Rivaroxaban, Pharmacokinetics, Pharmacodynamics

## Abstract

**Background:**

Novel biomarkers for personalizing anticoagulation remain undetermined. We aimed to investigate the association of plasma miRNAs with pharmacokinetic–pharmacodynamic (PK-PD) profiles of rivaroxaban.

**Methods:**

This is a multicenter, exploratory study of miRNAs in a Chinese population. Healthy volunteers and patients receiving rivaroxaban were enrolled in the study. The area under the plasma concentration–time curve from time 0-t h (AUC_0-t_) and anti-Xa activity at 3 h (AXA_3h_) were measured in healthy volunteers, and AXA_3h_ was measured in patients. MiRNAs were detected by miRNA microarray in 26 healthy volunteers with 20 mg rivaroxaban, and quantitative reverse transcription polymerase chain reaction was used to exclude undetectable ones. MiR-320a-3p and miR-483-5p were then quantified in 65 healthy volunteers and 71 patients. MiRNA levels at 3 h were compared between high and low AXA_3h_ or AUC_0-t_ subjects and in matched patients with or without bleeding during follow-up. The miRNA targets were predicted by TargetScan, miRTarBase, and miRDB. Validated genes were included in GO enrichment and KEGG analyses. The protein–protein interaction network was established by STRING and visualized by Cytoscape.

**Results:**

A total of 136 Chinese subjects completed the study. In healthy volunteers taking 15 mg rivaroxaban, the miR-320a level at 3 h was significantly positively correlated with AXA_3h_ and AUC_0-t_ (*r* = 0.359, *p* = 0.025; *r* = 0.370, *p* = 0.02, respectively). A positive correlation was also observed between miR-483 and AXA_3h_ or AUC_0-t_ (*r* = 0.372, *p* = 0.02; *r* = 0.523, *p* = 0.001, respectively). MiR-320a and miR-483 levels at 3 h in the higher AUC_0-t_ group were significantly higher than those at 0 h. MiR-483 levels at 3 h may distinguish healthy volunteers with high or low AXA_3h_ or AUC_0-t_. In the 10 mg fed subgroup, higher 3 h mir-483 levels were also observed compared with the control group. No significant differences were found in the comparisons among patients. Bioinformatic analysis showed that these miRNAs may play a regulatory role by targeting ABCG2, ITGB3, PTEN, MAPK1/3, etc.

**Conclusions:**

MiR-320a and miR-483 levels were found to be associated with PK and PD profiles of rivaroxaban in healthy Chinese subjects. Further studies are required to verify these findings and explore the mechanisms.

**Supplementary Information:**

The online version contains supplementary material available at 10.1186/s40246-022-00445-5.

## Background

Thromboembolic diseases are widespread and associated with high morbidity and mortality rates. Therefore, the prevention and treatment of thromboembolic disorders is of great importance. The routine use of direct oral anticoagulants (DOACs) is a major breakthrough in the management of thromboembolic diseases [1]. Compared with vitamin K antagonists, DOACs have predictable pharmacokinetic (PK) and pharmacodynamic (PD) profiles, low potential for drug interaction, and are administered at fixed doses without the need for regular monitoring [2].

Rivaroxaban, an oral direct factor Xa inhibitor, is a DOAC that has been approved for clinical use in several thromboembolic disorders, such as stroke prevention in patients with nonvalvular atrial fibrillation and prevention or treatment of venous thromboembolism (VTE) [3]. Rivaroxaban is absorbed rapidly, with maximum concentrations (*C*_max_) appearing 2 to 4 h after intake. The maximum factor Xa (FXa) inhibition is also reached 3 h after dosing [4, 5]. Oral bioavailability is high (80–100%) for the 10 mg tablet and is not affected by food. However, 15 mg and 20 mg tablets should be taken with food since coadministration with food can increase the area under the plasma concentration–time curve (AUC) and *C*_max_ [6]. A number of studies have confirmed that anti-Factor Xa chromogenic assays calibrated by rivaroxaban are best suited for the quantitative measurement of rivaroxaban plasma levels [7].

However, inter-individual variability in pharmacokinetic and pharmacodynamic parameters has been reported in healthy subjects and patients taking rivaroxaban [8, 9]. Changes in pharmacokinetics are expected to alter pharmacodynamics, which can cause drug toxicity or therapeutic failure. Therefore, biomarkers that can predict the PK and PD properties of rivaroxaban could help achieve the desired therapeutic drug effects and avoid bleeding in clinical drug therapy. Many genetic and non-genetic factors have been studied to determine whether they could contribute to this variability. However, the influence of genetic factors on clinical significance is inconsistent and based on limited research [10]. Moreover, biomarkers for predicting the PK and PD parameters of rivaroxaban are still lacking.

MicroRNAs (miRNAs), which are small non-coding RNAs with average lengths of 22 nucleotides, exist stably in various biofluids, including serum, plasma, and urine [11], and act as gene regulators [12]. A variety of miRNAs play a role in thrombosis and may act as biomarkers for thrombotic disease and a wide range of cardiovascular diseases such as atrial fibrillation, myocardial infarction, heart failure, atherosclerosis, hypertension, and type 2 diabetes mellitus [13–15]. It is also well recognized that miRNAs could be significant factors in the regulation of drug transporters and drug-metabolizing enzymes, and they may affect inter- and intra-individual variations in drug metabolism and disposition [16, 17]. Therefore, miRNAs may be associated with the PK-PD profiles of rivaroxaban, which are metabolized by several cytochrome P450 enzymes (CYP3A4/5, CYP2J2) and excreted by P-glycoprotein (P-gp) and breast cancer resistance protein (BCRP) [6]. The levels of miRNAs may have the potential to distinguish subjects with different drug responses and help personalize pharmacotherapy. To date, no study has explored the association between miRNAs and PK-PD profiles of DOACs, which may be important and instructive. Thus, the purpose of this study was to investigate the correlation between plasma miRNAs and PK-PD profiles of rivaroxaban in healthy subjects and to determine their relationship in patients.

## Methods

### Settings and ethics statement

This is a multicenter, exploratory study of miRNAs in a Chinese population. Healthy volunteers and patients receiving rivaroxaban were enrolled in the study. The inclusion criteria are listed in the Additional file 1: Table S1. The study was conducted in accordance with the Good Clinical Practice guidelines and the Declaration of Helsinki. The protocol was approved by an independent ethics committee, the Institutional Review Board of Peking University First Hospital, and all participating research sub-central hospitals. All subjects signed an informed consent form. The trial registration number is NCT03161496.

Healthy volunteers received a single dose of rivaroxaban (10, 15, or 20 mg) under either fasted or fed conditions. In the fasted state, rivaroxaban was administered with water in the morning following an overnight fast (10 h). In the fed state, rivaroxaban was administered within 30 min after consumption of a standard high-fat, high-calorie breakfast.

Baseline characteristics of the patients were recorded when they were enrolled. The incidence of bleeding events was recorded at 1, 6, and 12 months after administration by telephone or at an outpatient clinic. If the patient was not followed up at 12 months, data were obtained using the last observation carried forward (LOCF) method.

### Plasma collection

PK parameters were detected only in healthy volunteers, while PD parameters were detected in both healthy volunteers and patients. To detect PK parameters, venous blood samples (6 mL) were collected in 3.0/4.0 mL heparinized or EDTA-K2 tubes pre-dose and at 16–19 time points after drug administration. Blood samples were centrifuged at 4 °C for 10 min at 3000*g* within 120 min of sampling. To detect PD parameters, blood samples were collected in 2.7 mL sodium citrate (3.2% v/v) tubes at baseline and 3 h after administration (which was considered the time to peak [18]). They were then centrifuged at room temperature for 15 min at 2500*g* within 60 min of sampling. To detect miRNA profiles, 6 mL of peripheral blood was collected in EDTA-2 K tubes at baseline and 3 h in healthy volunteers, and at 3 h in patients. The samples were centrifuged at 3000*g* and 4 °C for 10 min.

All plasma samples were immediately stored at −80 °C until analysis. PK assessment was conducted in each sub-center, and no significant matrix effect was found. Detailed PK assessment methods are shown in Additional file 2: Table S2. PD and miRNA assessments were performed at a centralized facility in the Peking University First Hospital.

### PK, and PD analysis

PK parameters were determined by standard non-compartmental methods using Phoenix WinNonlin 7.0, (Pharsight Corporation, Sunnyvale, CA, USA) or DAS for EDC V5.0 (Medbanks, Beijing, China). The AUC from time 0-t h (AUC_0-t_) was calculated using the linear trapezoidal rule.

The PD parameter, anti-FXa activity (AXA) of rivaroxaban, was measured using a validated chromogenic anti-FXa kit (BIOPHEN DiXaI®, HYPHEN BioMed, Neuville sur Oise, France) with rivaroxaban calibrators and controls (BIOPHEN Rivaroxaban® Calibrator and Control, HYPHEN BioMed, Neuville sur Oise, France). AXA levels were determined using a Sysmex® CS2100i (Sysmex, Kobe, Japan) instrument with a validated application.

### Study design

**Fig. 1:**
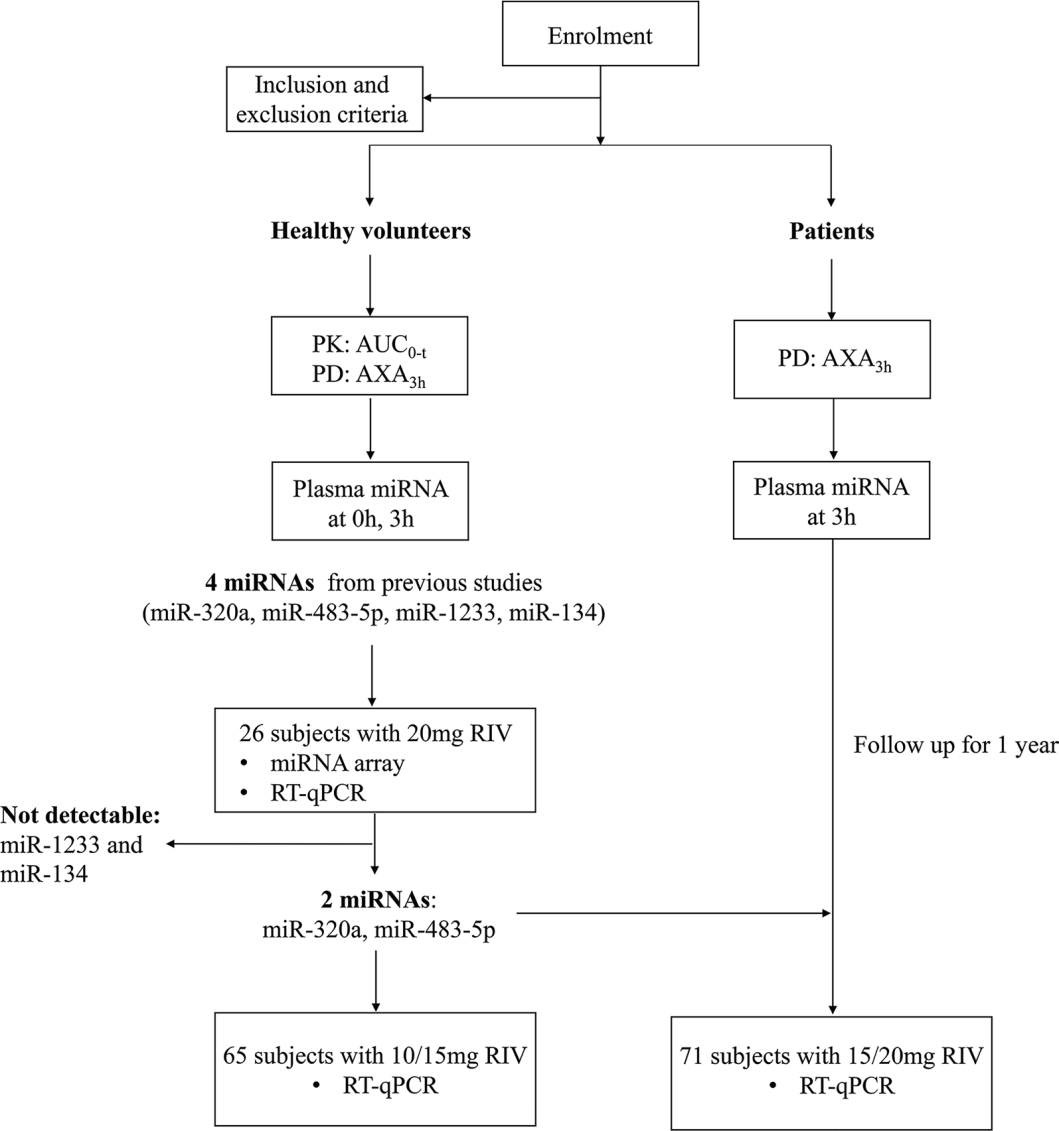
Flowchart of the technological process. PK, pharmacokinetic profiles; PD, pharmacodynamic profiles; AXA_3h_, anti-Xa activity measured 3 h after rivaroxaban administration; AUC_0-t_: the area under the plasma concentration–time curve from time 0-t h; RT-qPCR: quantitative reverse transcription polymerase chain reaction; RIV: rivaroxaban

Based on previous literature, we focused on four candidate miRNAs (miR-320a, miR-483-5p, miR-1233, and miR-134) that are associated with cardiovascular and thromboembolic diseases [13, 19, 20]. We used a microarray that contained miRNAs of interest to detect their levels in 26 healthy volunteers with 20 mg rivaroxaban. Subsequently, quantitative reverse transcription polymerase chain reaction (RT-qPCR) was conducted on the same samples to exclude undetectable miRNAs. Finally, miR-320a and miR-483-5p were selected and validated by RT-qPCR in 65 healthy volunteers with 10 or 15 mg rivaroxaban and 71 patients with 15 or 20 mg rivaroxaban. Figure 1 shows a general flowchart of the study.

Since different doses and diet status affect the pharmacokinetic parameters of rivaroxaban [18], healthy volunteers were classified by dose and as fasted or fed. In the discovery phase, subjects with high or low AXA_3h_ values were classified into case and control groups, respectively. In the validation phase, the top ~ 40% of subjects with high AXA_3h_ or AUC_0-t_ values in each dosage group were classified as the case group, while the bottom ~ 40% of subjects with low values were classified as the control group. The miRNAs of the subjects with relatively high or low values were compared. Patients were classified into case and control groups according to the AXA_3h_ value. The top and bottom ~ 20% of the patients with high or low AXA_3h_ were treated as the case and control groups, respectively. Moreover, the patients were classified according to the occurrence of bleeding events within 12 months. Case–control matching was used in patients with or without bleeding based on dose, age (± 3 years), sex, diet, indication, whether performed radiofrequency catheter ablation (RFCA), and whether combined with coronary heart disease.

### miRNA microarray

Agilent miRNA array was used for the miRNA profiling in accordance with the manufacturer's instructions. Each slide of the Agilent array has eight identical arrays (8 × 60 K format), and each array contains probes that interrogates 2549 human mature miRNAs from miRBase R21.0. Each miRNA was detected by probes repeat for 30 times. 2164 Agilent control probes are also included in the array. In a nutshell, the Agilent miRNA labeling reagent was used to label the miRNAs. The labeled RNA was isolated and hybridized to miRNA arrays after being dephosphorylated and ligated with pCp-Cy3 using 200 ng of total RNA. Agilent feature extraction software version 10.10 was used to analyze the images after they had been scanned using the Agilent microarray scanner (Agilent). The GeneSpring software V13 (Agilent) was used to analyze the miRNA array data for data summarization, standardization, and quality control (Agilent).

### Quantitative reverse transcription polymerase chain reaction

Total RNA was isolated from 200 µL of plasma using the miRNeasy Serum/Plasma Advanced Kit (QIAGEN). Reverse transcription was performed using the TaqMan Advanced miRNA cDNA synthesis kit (Thermo Fisher Scientific). Subsequently, RT-qPCR reactions were performed using the predesigned TaqMan Advanced miRNA Assays (Thermo Fisher Scientific) on the StepOne Plus Real-Time PCR System (Applied Biosystems). All procedures were performed according to the manufacturer’s instructions. The expression of miR-16-5p is stable according to the literature, and it is often used as an endogenous reference [21–25]. Therefore, the relative expression levels of miRNAs were normalized to has-miR-16-5p. All RT-qPCR experiments were performed in triplicate, and the mean Ct values were calculated. The relative expression of miRNA was evaluated using the comparative cycle threshold (Ct) method and reported as 2^−ΔCt^, where ΔCt = mean Ct_miRNA of interest_-mean Ct_miR-16_.

### Bioinformatics analysis

The miRNA-predicted targets were analyzed using three different algorithms (TargetScan 8.0, miRTarBase 8.0, and miRDB 6.0) [26–28]. Only target genes validated by qPCR, western blotting, or reporter assays were included in the following analysis. Gene ontology (GO) enrichment and Kyoto Encyclopedia of Genes and Genomes (KEGG) analyses of the predicted genes were performed using Metascape (http://metascape.org) [29]. The default value was used as the cut-off criterion.

The miRNA–mRNA regulatory network was visualized by Cytoscape 3.7.2 [30]. The protein–protein interaction (PPI) network of the predicted target genes of the two miRNAs was established through STRING 11.5 (Search Tool for the Retrieval of Interacting Genes database (STRING) website (http://string-db.org) [31]. The disconnected nodes were hidden in the network. The network was visualized by Cytoscape 3.7.2 and further analyzed using molecular complex detection (MCODE) [32] and CytoHubba [33]. The MCODE analysis is a density-based non-overlapping clustering algorithm. Hub genes were identified using CytoHubba software. All parameters of the plugin were left at their default values.

### Statistical analysis

Demographic and clinical characteristics are presented as median ± interquartile range (IQR) for continuous variables and compared using the Mann–Whitney test. Categorical variables were presented using count (frequency) and tested for baseline comparability using Fisher's exact test. Differences in miRNA levels between the case and control groups were evaluated using the Mann–Whitney unpaired test. Wilcoxon matched-pairs signed rank test was used for comparison between 0 and 3 h samples in the same group. Spearman correlation analysis was performed to describe the correlation. Univariate logistic regression analysis was performed, presenting the odds ratio (OR) and the 95% confidence interval (CI). The area under the curve (AUC) of the receiver operating characteristic curve was used to assess the predictive accuracy of miRNAs. Statistical analysis and graphics were performed using the Statistical Package for Social Sciences (SPSS version 24.0, SPSS Inc., Chicago, IL, USA) and GraphPad Prism 9. Two-sided tests where *p* < 0.05 were considered statistically significant.

## Results

### Recruitment and participant characteristics

Twenty-Six healthy volunteers taking 20 mg rivaroxaban with high or low AXA_3h_ were selected for miRNA microarray analysis. RT-qPCR was conducted on the same samples. Since miR-1233 and miR-134 were not detectable in at least 90% of the individuals after RT-qPCR, we excluded these two miRNAs for further analysis, and miR-320a and miR-483-5p were used.

MiR-320a and miR-483-5p were quantified by RT-qPCR in 65 healthy volunteers (10 mg, *n* = 26; 15 mg, *n* = 39). The baseline characteristics are shown in Table 1. Significant differences in AXA_3h_ and AUC_0-t_ were observed between the case and control groups. There were no significant differences in sex, age, or body mass index (BMI) between the two groups.

**Table 1:** Baseline characteristics of healthy volunteers whose miRNA levels were quantified

	Total	Group classified by AXA_3h_				Group classified by AUC_0-t_			
		Overall	Case	Control	*p* value	Overall	Case	Control	*p* value
10 mg									
* n*	26	20	10	10	1.000	20	10	10	1.000
Male	15 (57.69%)	11 (55.00%)	4 (40.00%)	7 (70.00%)	0.370	12 (60.00%)	8 (80.00%)	4 (40.00%)	0.170
Fed	13 (50.00%)	10 (50.00%)	5 (50.00%)	5 (50.00%)	1.000	10 (50.00%)	5 (50.00%)	5 (50.00%)	1.000
Age	32 (13)	34 (16)	37.5 (16)	31.5 (14)	0.579	35.5 (15)	33 (14)	36 (14)	0.796
BMI	23.35 (1.8)	23.35 (1.70)	23.40 (2.50)	22.60 (1.60)	0.853	23.40 (2.00)	23.10 (2.10)	23.40 (1.80)	0.912
AXA_3h_	134.04 (70.98)	134.58 (123.28)	195.93 (74.51)	86.28 (32.26)	0.000*	117.08 (78.49)	92.95 (35.58)	167.41 (64.05)	0.000*
*C*_3_	163.1745 (108.41)	160.95 (104.41)	197.43 (85.37)	103.34 (45.57)	0.000*	146.36 (99.96)	103.34 (42.82)	194.46 (62.13)	0.000*
*C*_max_	191.9635 (69.81)	188.00 (96.67)	225.14 (78.56)	137.44 (78.92)	0.003*	187.16 (82.93)	137.44 (67.60)	215.98 (57.62)	0.001*
AUC_0-t_	1302.5875 (751.46)	1231.48 (780.15)	1633.65 (622.65)	910.76 (415.46)	0.000*	1350.74 (866.89)	840.75 (354.44)	1702.33 (316.65)	0.000*
15 mg									
*n*	39	28	14	14	1.000	28	14	14	1.000
Male	33 (84.62%)	23 (82.14%)	10 (71.43%)	13 (92.86%)	0.326	23 (82.14%)	13 (92.86%)	10 (71.43%)	0.326
Fed	21 (53.85%)	14 (50.00%)	7 (50.00%)	7 (50.00%)	1.000	14 (50.00%)	7 (50.00%)	7 (50.00%)	1.000
Age	29 (10)	27 (11)	24 (8)	30 (11)	0.114	25 (9)	27 (10)	24.5 (9)	0.401
BMI	22.2 (3.2)	22.10 (2.70)	21.70 (2.30)	23.20 (2.90)	0.401	23.10 (3.30)	23.30 (3.50)	22.55 (3.40)	0.804
AXA_3h_	171.22 (175.59)	164.39 (219.13)	273.28 (79.31)	54.78 (113.13)	0.000*	169.96 (155.77)	93.07 (109.99)	221.93 (75.05)	0.000*
*C*_3_	208 (183)	210.00 (224.25)	329.50 (75.75)	107.00 (75.75)	0.000*	203.00 (169.50)	129.00 (99.00)	277.00 (100.75)	0.000*
*C*_max_	283 (164)	284.00 (172.75)	358.50 (53.75)	196.00 (78.75)	0.000*	283.00 (136.25)	201.50 (86.25)	326.00 (86.50)	0.000*
AUC_0-t_	1782.71 (1212.23)	1816.22 (1123.77)	2118.18 (542.33)	1090.19 (491.24)	0.000*	1728.31 (1402.69)	1074.30 (468.58)	2440.31 (653.37)	0.000*

Values are median (IQR), *n* (%)

*IQR* interquartile range; *AXA*_*3h*_ anti-Xa activity measured 3 h after rivaroxaban administration, *AUC*_*0-t*_ area under the plasma concentration time-curve from time 0 to time of last determinable concentration, *BMI* body mass index, C_3_ plasma concentration 3 h after administration, *C*_*max*_ maximum plasma concentration

*p < 0.05

In the patient group, miRNAs from 71 patients taking 15 or 20 mg rivaroxaban in two sub-centers were quantified. Most patients took rivaroxaban for atrial fibrillation (AF) (97.2%). 25 patients (35.2%) took 15 mg and 46 patients (64.8%) took 20 mg. There were significant differences in AXA_3h_ between the case and control groups. 23 patients suffered any bleeding events within their follow-up. Six pairs of patients were matched based on the occurrence of bleeding events within one year and demographic characteristics. The baseline characteristics of the patients are presented in Table 2.

**Table 2:** Baseline characteristics of the patients

	Overall (*n* = 71)
Dosage	
* n* (15 mg)	25 (35.2%)
* n* (20 mg)	46 (64.8%)
Diet status (fasted/fed)	45/26
Male	35 (49.3%)
Age, years	68 (15)
BMI, kg/m^2^	26.26 (4.05)
Indication	
AF	69 (97.2%)
VTE	4 (5.6%)
RFCA	35 (49.3%)
AXA_3h_	348.66 (156.21)
Case (*n* = 14)	508.28 (74.05)
Control (*n* = 14)	202.11 (76.66)
*p* value	0.000*
Smoking (yes/never/cessation)	10/54/7
Comorbidity	
Diabetes	17 (23.9%)
Hyperlipemia	23 (32.4%)
Coronary heart disease	29 (40.8%)
Baseline blood tests	
HGB, g/L	133 (24)
PLT, 10^9^/L	188 (65)
MPV, fL	9.1 (1.62)
Outcomes	
Any bleeding	23 (32.4%)

Values are median (IQR), *n* (%)

*IQR* interquartile range, *BMI* body mass index, *AF* atrial fibrillation, *VTE* venous thromboembolism, *RFCA* radiofrequency catheter ablation, *HGB* hemoglobin, *PLT* platelet, *MPV* mean platelet volume

*p < 0.05

### Association between miRNAs at 3 h and rivaroxaban PK-PD profiles

#### Healthy volunteers with 15 mg rivaroxaban

**Fig. 2:**
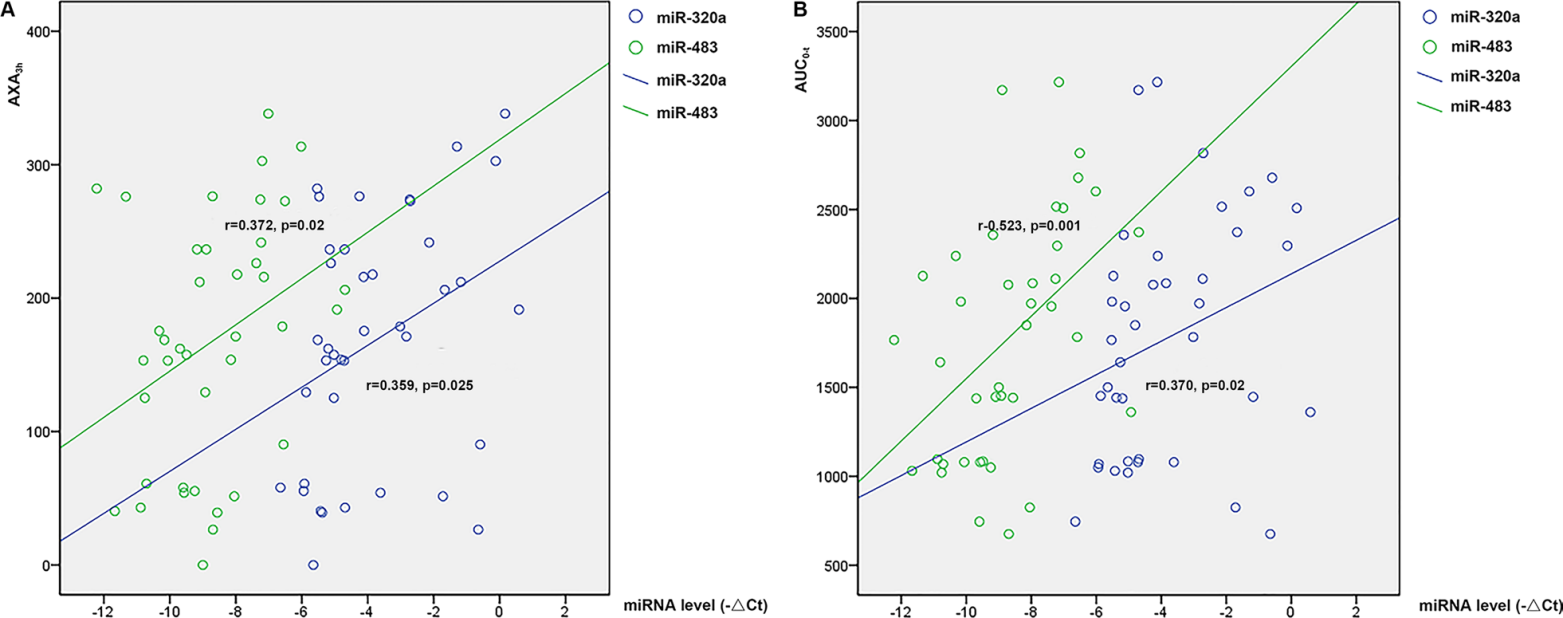
Correlation between two miRNAs and AXA_3h_ (**A**) or AUC_0-t_ (**B**). AXA_3h_, anti-Xa activity measured 3 h after rivaroxaban administration; AUC_0-t_: the area under the plasma concentration–time curve from time 0-t h

Among the thirty-nine healthy volunteers taking 15 mg rivaroxaban whose miRNA levels were detected, AXA_3h_ was significantly positively correlated with AUC_0-t_ (*r* = 0.739, *p* = 0.000). The miR-320a level at 3 h was significantly positively correlated with AXA_3h_ and AUC_0-t_ (*r* = 0.359, *p* = 0.025; *r* = 0.370, *p* = 0.02, respectively). A positive correlation was also observed between miR-483 and AXA_3h_ or AUC_0-t_ (*r* = 0.372, *p* = 0.02; *r* = 0.523, *p* = 0.001, respectively). Figure 2 shows the correlation between these two miRNAs and AXA_3h_ (A) and AUC_0-t_ (B).

For miR-320a, there was no significant difference between the case and control groups, regardless of whether AXA_3h_ or AUC_0-t_ was used to distinguish between the two groups. No significant differences were found in the subgroups classified by diet status. For miR-483, the plasma level was significantly higher in the case group than in the control group (group classified by AXA_3h_, *p* = 0.0310; group classified by AUC_0-t_, *p* = 0.0067). After analysis by diet status, significant differences were found between the two fasted groups (group classified by AXA_3h_, *p* = 0.0111; group classified by AUC_0-t_, *p* = 0.0379), but no significant difference was observed in the fed group. The significant association between miRNAs at 3 h and rivaroxaban PK-PD profiles are summarized in Table 3 and Additional file 3: Table S3.

**Table 3:** Significant association between miRNAs at 3 h and rivaroxaban PK-PD profiles

Group criteria	Fasted	FC_case–control_	*p* value	Fed	FC_case–control_	*p*	Total	FC_case–control_	*p* value
**15 mg**									
Grouped by AXA_3h_									
3 h: case vs control	miR-483↑	3.38	0.0111	–	–	–	miR-483↑	2.44	0.0310
Grouped by AUC_0-t_									
3 h: case vs control	miR-483↑	3.12	0.0379	–	–	–	miR-483↑	2.21	0.0067
**10 mg**									
Grouped by AXA_3h_									
3 h: case vs control	–	–	–	miR-483↑	213.95	0.0317	–	–	–
Grouped by AUC_0-t_									
3 h: case vs control	–	–	–	miR-320a↑	35.89	0.0317	–	–	–
3 h: case vs control	–	–	–	miR-483↑	191.69	0.0159	–	–	–

↑ indicates that this miRNA expression at 3 h was significantly higher in the case group than in the control group

–: None

*FC*_*case–control*_ fold change between case and control

Logistic regression analysis was used to further assess the association between miRNAs and PK-PD profiles. Since miRNA levels (2^−△Ct^) were non-normally distributed and the clinical significance of increasing each additional unit was not clear, we scaled the two miRNAs by their respective IQR [34] to examine the effect of miRNAs on the risk for high AXA_3h_, as estimated by odds ratios. The results showed that the miR-483 level at 3 h standardized by IQR could significantly differentiate the case and control groups classified by AXA_3h_ (OR, 5.608; 95% CI, 1.112–28.282; *p* = 0.037). The same analysis was performed to identify the indicators that differentiated groups classified by AUC_0-t_. However, miR-320a and miR-483 levels did not differ between the two groups (Additional file 4: Table S4).

**Fig. 3:**
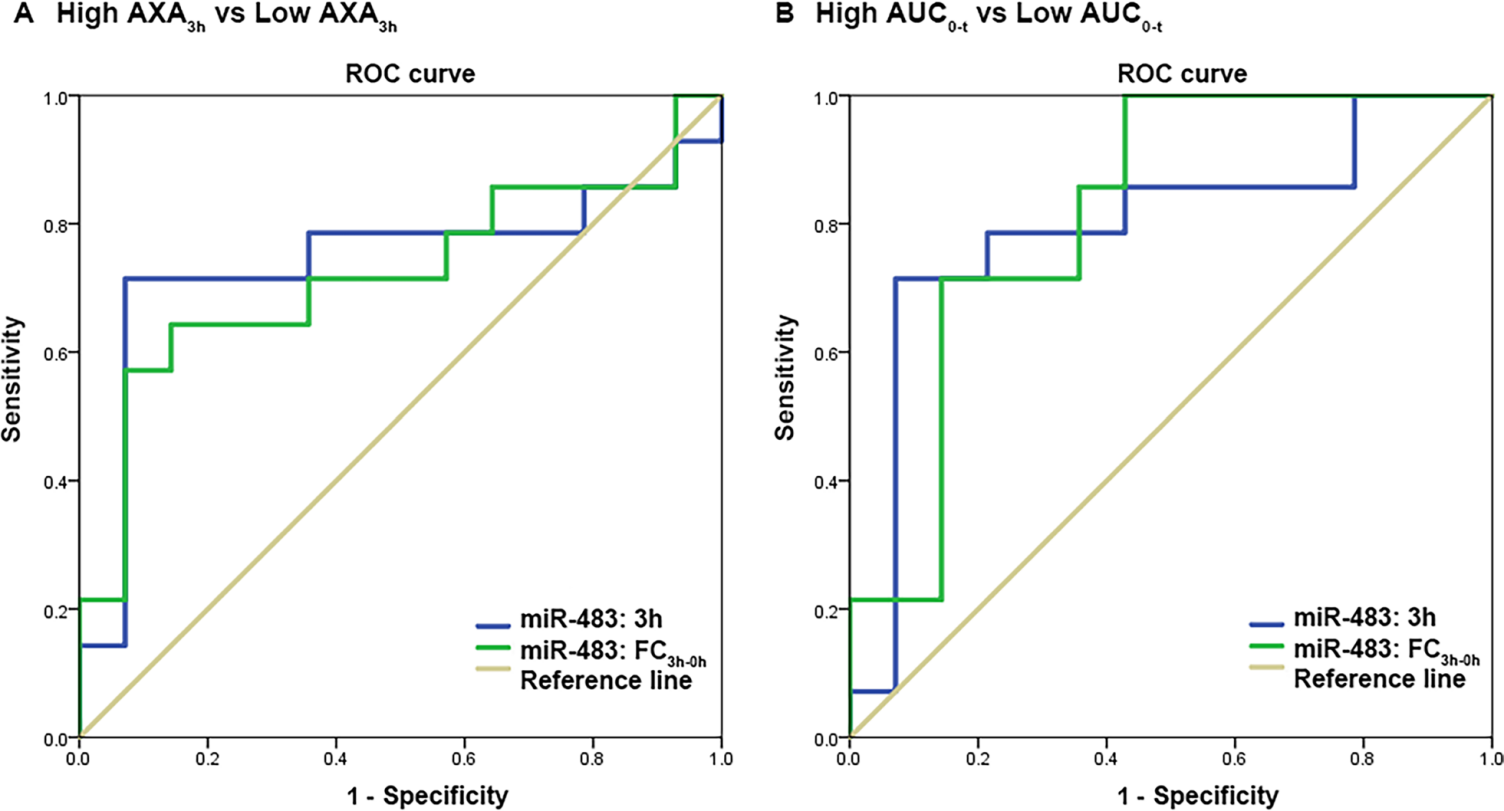
ROC curve of 3 h miR-483 level and FC_3h-0h_ in case and control healthy volunteers taken 15 mg rivaroxaban. **A** group classified by AXA_3h_; **B** group classified by AUC_0-t_. AXA_3h_, anti-Xa activity measured 3 h after rivaroxaban administration; AUC_0-t_: the area under the plasma concentration–time curve from time 0-t h; FC_3h-0h_: fold change between 3 and 0 h

To estimate the predictive value of miR-483 for indicating the rivaroxaban response, ROC curves and AUCs were calculated. In healthy volunteers taking 15 mg rivaroxaban, ROC analysis revealed an AUC of miR-483 levels (represented by 2^−△Ct^) in the groups classified by AXA_3h_ and AUC_0-t_ of 0.740 (95% CI = 0.533–0.946; sensitivity: 71.4%; specificity: 92.9%; *p* = 0.031) and 0.796 (95% CI = 0.616–0.976; sensitivity: 71.4%; specificity: 92.9%; *p* = 0.008), respectively, when a cut-off value of 0.00384 was used (Fig. 3).

#### Healthy volunteers with 10 mg rivaroxaban

Among twenty-six healthy volunteers who took 10 mg rivaroxaban, AXA_3h_ was significantly positively correlated with AUC_0-t_ (*r* = 0.816, *p* = 0.000). MiR-320a and miR-483 level at 3 h were also positively correlated with AXA_3h_ and AUC_0-t_; however, this was not significant. In the fed subgroup, miR-320a level at 3 h in the case group (classified by AUC_0-t_) was significantly higher than that in the control group (*p* = 0.0317). MiR-483 level at 3 h in the fed case group was also significantly higher than that in the control group (group classified by AXA_3h_, *p* = 0.0317; group classified by AUC_0-t_, *p* = 0.0159), as shown in Table 3 and Additional file 3: Table S3. Odds ratio analysis showed that neither miRNA could differentiate between the two groups (Additional file 4: Table S4).

#### Patients with 15 or 20 mg rivaroxaban

In 71 patients, we did not find a significant association between AXA and bleeding events. For miR-320a and miR-483, the comparison between the case and control groups showed no significant differences. There was also no significant difference between the matched patients with and without bleeding (Additional file 5: Table S5). Neither miR-320a nor miR-483 was a predictive factor that could distinguish between case and control groups classified by AXA_3h_ according to odds ratio analysis. Among matched bleeding and non-bleeding patients, miR-320a and miR-483 levels could not differentiate the risk of bleeding events (Additional file 6: Table S6).

### Association between miRNAs dynamics and rivaroxaban PK-PD profiles

In healthy volunteers administered 15 mg rivaroxaban, spearman correlation analysis showed that the fold change of miR-320a between 3 and 0 h (FC_3h-0h_) was significantly positively correlated with AUC_0-t_ (*r* = 0.319, *p* = 0.048), and the FC_3h-0h_ of miR-483 was significantly positively correlated with AUC_0-t_ (*r* = 0.394, *p* = 0.013). The FC_3h-0h_ of these two miRNAs were also positively correlated with AXA_3h_ but were not statistically significant.

**Fig. 4:**
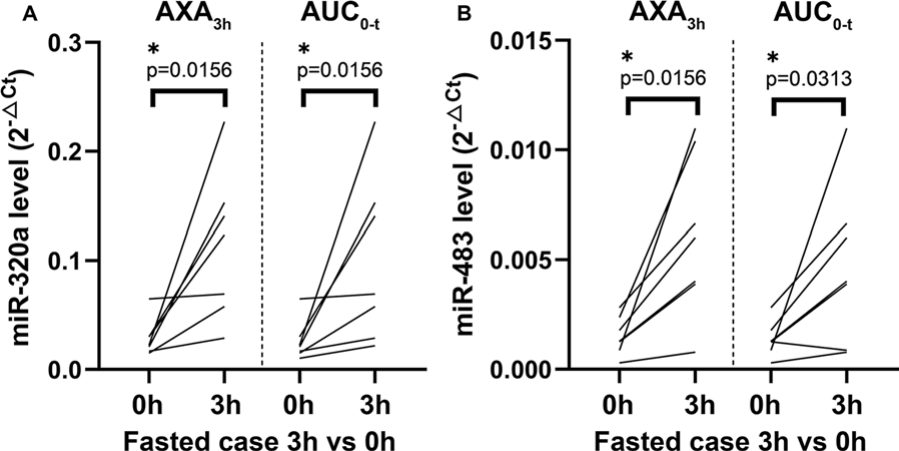
Comparison of miR-320a (**A**) and miR-483 (**B**) level between 3 and 0 h in the 15 mg fasted case group

The miRNA levels were compared at different time points in the same group, and the miR-320a level at 3 h was significantly higher than that at 0 h in the case group (group classified by AXA_3h_, *p* = 0.0085; group classified by AUC_0-t_, *p* = 0.0001). Among fasted subjects, significant differences were also found between 3 and 0 h in the case group (group classified by AXA_3h_, *p* = 0.0156; group classified by AUC_0-t_, *p* = 0.0156) (Fig. 4A). In fed subjects, a significant difference was found between 3 and 0 h in the case group classified by AUC_0-t_. However, no significant differences were observed in miR-320a levels between the different time points in the control group. For miR-483, the level at 3 h was significantly higher than 0 h in the case group classified by AUC_0-t_ (*p* = 0.0017). When subgroup analysis was conducted in fasted subjects, significant differences were found between 3 and 0 h in the case group, regardless of the classification method used (group classified by AXA_3h_, *p* = 0.0156; group classified by AUC_0-t_, *p* = 0.0313) (Table 4, Fig. 4B). There was no significant difference between the two time points in the control group. In healthy volunteers with 10 mg rivaroxaban, no significant differences in miR-320a or miR-483 levels were found between 3 and 0 h in the case or control group; besides, since miRNA at baseline was not collected in patients, a comparison between 3 and 0 h cannot be conducted. The associations between miRNA dynamics and rivaroxaban PK-PD profiles are summarized in Table 4 and Additional file 7: Table S7.

**Table 4:** Association between miRNAs dynamics and rivaroxaban PK-PD profiles

Group criteria	Fasted	FC_3h-0h_	*p* value	Fed	FC_3h-0h_	*p* value	Total	FC_3h-0h_	*p* value
**15 mg**									
Grouped by AXA_3h_									
Case: 3 h vs 0 h	miR-320a↑	3.99	0.0156	–	–	–	miR-320a↑	5.47	0.0085
	miR-483↑	4.01	0.0156	–	–	–	–	–	–
Grouped by AUC_0-t_									
Case: 3 h vs 0 h	miR-320a↑	3.86	0.0156	miR-320a↑	7.87	0.0156	miR-320a↑	6.46	0.0001
	miR-483↑	3.48	0.0313	–	–	–	miR-483↑	3.98	0.0017

↑ indicates that this miRNA expression in the case group was significantly higher at 3 h than at 0 h

–: None

*FC*_*3h-0h*_ fold change between 3 and 0 h

In healthy volunteers with 15 mg rivaroxaban, logistic regression analysis showed that miR-320a could not distinguish between the case and control groups. In the fasted group, FC_3h-0h_ of miR-483 may be a predictor of high or low AUC_0-t_ (OR, 6.177; 95% CI 1.04–36.696; *p* = 0.045). ROC analysis showed that the AUC of FC_3h-0h_ of miR-483 in the groups classified by AXA_3h_ and AUC_0-t_ was 0.719 (95% CI = 0.519–0.920; sensitivity: 64.3%; specificity: 85.7%; *p* = 0.048), 0.816 (95% CI = 0.652–0.981; sensitivity: 71.4%; specificity: 85.7%; *p* = 0.004), respectively, when a cut-off value of 2.184 was used (Fig. 3).

In healthy volunteers with 10 mg rivaroxaban, no significant difference in miR-320a or miR-483 was found between 3 and 0 h in the case group, although an upward trend was observed (Additional file 7: Table S7).

### Bioinformatics analysis for miRNA target genes and biological pathways

Three target prediction databases were used to identify the target genes of miR-320a and miR-483 (Additional file 8: Table S8). According to the miRTarBase 8.0, 34 validated genes of two miRNAs were obtained. The results showed that these miRNAs potentially regulated important genes, such as those encoding integrin subunit beta 3 (ITGB3), phosphatase and tensin homolog (PTEN), and mitogen-activated protein kinase 1/3 (MAPK1/3).

**Fig. 5:**
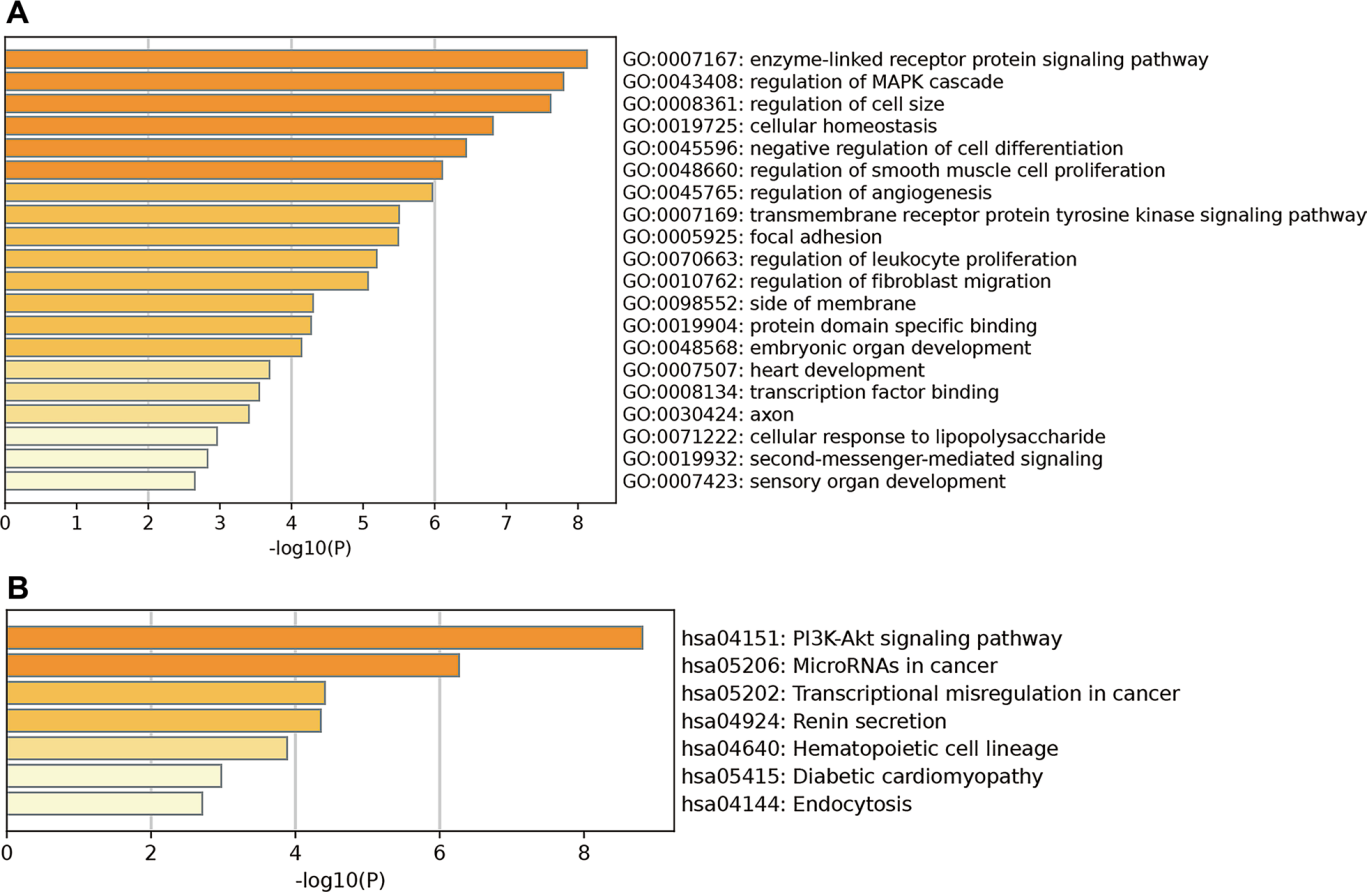
Bioinformatic analysis of predicted targets of two miRNAs performed by Metascape. **A** Top 20 GO pathways analysis; **B** Top 7 KEGG pathways analysis. GO: gene ontology; KEGG: Kyoto Encyclopedia of Genes and Genomes

Subsequently, the validated genes were subjected to GO and KEGG analyses using Metascape. The top 20 clusters with their representative enriched terms for GO analysis are shown in Fig. 5A. The target genes of miR-320a-3p were significantly enriched in enzyme-linked receptor protein signaling pathway, regulation of MAPK cascade, regulation of cell size, cellular homeostasis, and negative regulation of cell differentiation. KEGG pathway analysis showed that the enrichment terms converged on the PI3K-Akt signaling pathway, microRNAs in cancer, transcriptional misregulation in cancer, renin secretion, and hematopoietic cell lineage, among others (Fig. 5B).

**Fig. 6:**
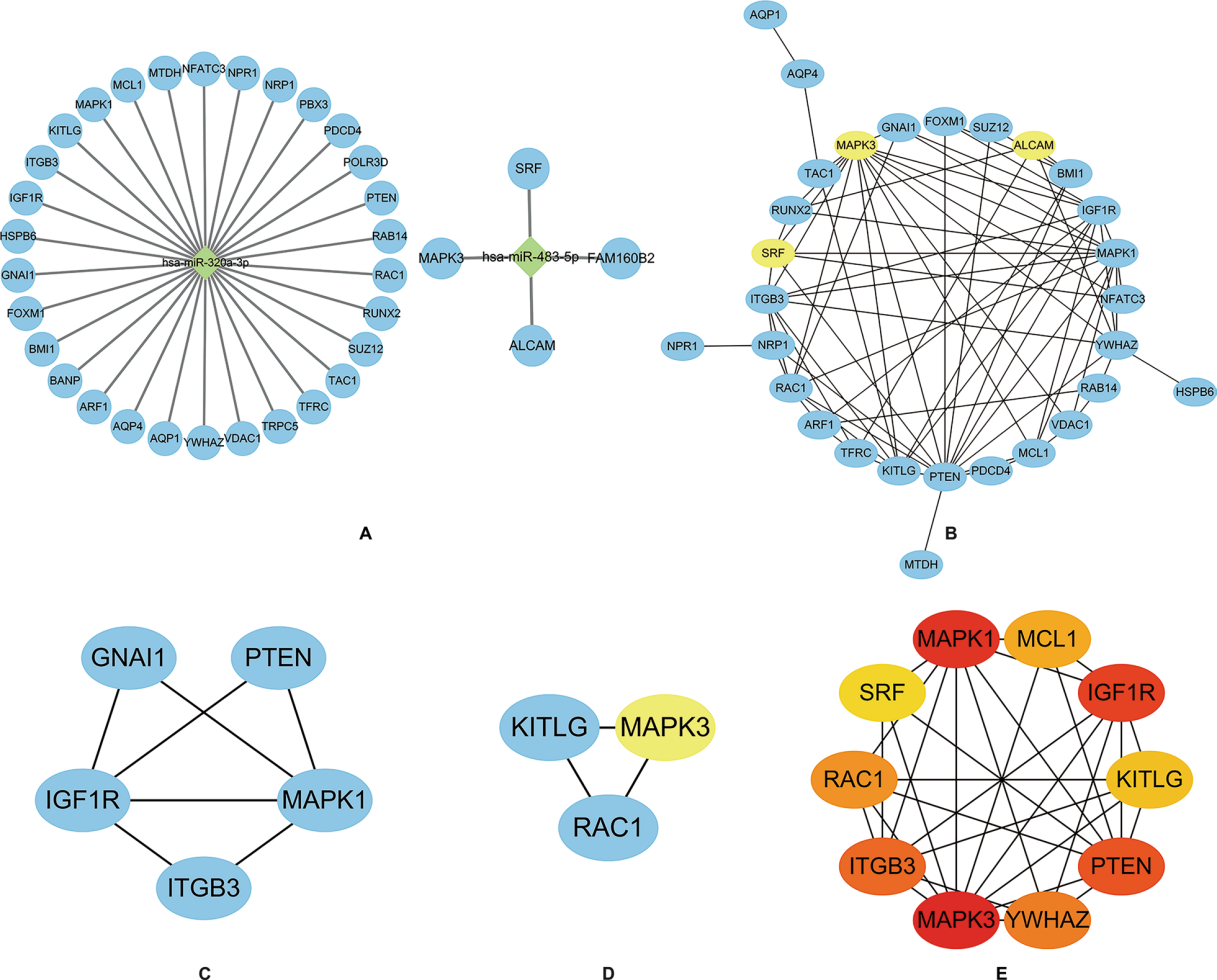
MiRNA-mRNA regulatory network, PPI network, top clusters, and hub genes analysis. **A** MiRNA-mRNA regulatory network; **B** PPI network of predicted target genes; **C**, **D** Top two clusters in MCODE analysis (Yellow nodes represent target genes of miR-483, while blue nodes represent genes of miR-320a); **E** Top 10 hub genes ranked by MCC method using CytoHubba (Dark nodes represent genes with high MCC scores, while light nodes represent genes with low MCC scores). MCODE: Molecular Complex Detection. MCC: Maximal Clique Centrality

A total of 34 validated target genes targeted by two miRNAs, were selected for construction of a miRNA-mRNA network (Fig. 6A). The PPI network had 29 nodes and 75 edges (Fig. 6B). We screened the top two clusters with the highest clustering scores using the MCODE plugin (Fig. 6C, D). Moreover, ten hub genes (MAPK3, MAPK1, IGF1R, PTEN, ITGB3, YWHAZ, RAC1, MCL1, KITLG, and SRF) were identified from the PPI network using the CytoHubba plugin of Cytoscape (Fig. 6E).

## Discussion

To the best of our knowledge, this is the first study to assess the relationship between miRNAs and PK-PD profiles of rivaroxaban in Chinese populations, providing a model for further clinical and mechanistic research on miRNAs.

Currently, data on miRNAs influencing PK/PD and clinical outcomes of rivaroxaban application remain unclear. Only one study assessed the role of miR-142 and miR-39 for drug-monitoring of rivaroxaban among elderly patients with atrial fibrillation [35]. However, the sample size was small and AXA activity was not analyzed. Due to the insufficient information on miRNAs in Chinese populations, we discover novel miRNA biomarkers and their respective values of influence on individual anticoagulation therapy with rivaroxaban.

This study found that in healthy volunteers taking 15 mg rivaroxaban, miR-320a and miR-483 measured at 3 h post-dose were significantly positively correlated with AXA_3h_ and AUC_0-t_. The level of miR-483 at 3 h in the case group was significantly higher than that in the control group. The miR-483 level standardized by IQR measured at 3 h was the only factor that could significantly differentiate case and control groups classified by AXA_3h_. In addition, ROC analysis showed that miR-483 level had the potential to distinguish groups classified by AXA_3h_ or AUC_3h_. Moreover, in the group with higher AUC_0-t_ values, miR-320a and miR-483 at 3 h were significantly higher than those at 0 h. Subgroup analysis showed that miR-320a and miR-483 at 3 h were significantly higher than that at 0 h in fasted groups with higher AXA_3h_ or AUC_0-t_. Additionally, ROC analysis showed that FC_3h-0h_ of miR-483 level also had the potential to distinguish groups classified by AXA_3h_ or AUC_3h_. At a dose of 10 mg, miR-483 at 3 h was significantly higher in the fed case group than in the control group, and miR-320a expression was significantly higher in the high AUC_0-t_ group than in the low group. No significant differences were found in the other comparisons. The insignificant effect between 3 and 0 h may be due to the smaller dosage size.

Furthermore, we measured the miRNA levels in patients. However, the results obtained in healthy people were not well validated in patients. The reasons for the failure to verify the effect of miRNAs on PD and bleeding events in patients could be as follows. First, multiple factors, including diseases, combined medication, renal impairment, hepatic impairment, age, body weight, gender, ethnicity, and diet, may influence the metabolism of the drug and these indexes are different between healthy subjects and patients. For rivaroxaban, age and renal function are most vital [6]. In this study, patients were significantly older than the healthy subjects, which may provide differences in PK profiles. Thus, the presence of a variety of confounding factors may lead to the cover-up of miRNA effects. Second, previous clinical studies had shown a correlation between AXA and bleeding events [36, 37], whereas no significant correlation was observed in our study. Furthermore, as an exploratory study, the sample size of healthy subjects and patients was small. Therefore, results need to be verified in the well-organized study which involved more patients, and subgroup analysis may help provide more information.

Bioinformatic analysis revealed that miR-320a and miR-483 regulate different genes and are involved in different pathways. The potential mechanism regulating the PK and PD profiles of rivaroxaban is discussed here.

1. The association of miR-320a, and BCRP (gene code ABCG2)

Drug-metabolizing enzymes, transporters, nuclear receptors, and transcription factors are vital for the absorption, distribution, metabolism, and excretion (ADME) of drugs. Knowledge of the mechanisms involved in the expression and function of ADME genes is critical for predicting drug efficacy and safety profiles and lays the foundation for future precision medicine [38–40]. Rivaroxaban, an oral, direct factor Xa inhibitor, is metabolized and excreted via CYP3A4/3A5/2J2, P-gp, and BCRP [6]. Approximately one-third of the dose is eliminated as unchanged active drug in the urine via a process thought to involve P-gp (gene code ABCB1) and BCRP (gene code ABCG2), which play important roles in the absorption of rivaroxaban, a class 2 drug according to the Biopharmaceutics Drug Disposition Classification System (BDDCS) classification system [41]. We hypothesized that miRNAs may act on transporter mRNA 3UTR or nuclear receptors, transcription factors, and other signaling molecules, which are transporter regulators.

In a previous study, miR-320a negatively regulated the expression of ABCG2 at the transcriptional level [42]. Kang et al. reported that miR-320a suppressed the promoter activity of ABCG2, but not the luciferase activity of its 3-UTR, and found that the promoter region of ABCG2 contained putative binding sites for nuclear factor of activated T cell isoform c3 (NFATc3). Thus, they speculated that miR-320a decreased the expression of ABCG2 at the transcriptional level by targeting NFATc3. In another study, miR-320a was validated to directly target the 3-UTR of NFATc3 [43]. They found that NFATC3 was essential for P-gp-induced chemoresistance. Therefore, we speculated that high levels of miR-320a reduce rivaroxaban transport activity by downregulating ABCG2 at the transcriptional level, leading to variability in PK and PD profiles (a high value of AUC_0-t_ and AXA_3h_).

2. The association of miR-320a, miR-483 and MAPK signaling pathway

Regulation of the MAPK cascade ranks high in the GO enrichment analysis. MAPK3 and MAPK1 are both hub genes ranked by the CytoHubba plugin, and they are predicted target genes of miR-483 and miR-320a, respectively, in all three algorithms. The MAPK cascade plays a critical role in the regulation of several fundamental processes, such as proliferation, differentiation, and cell response to diverse extrinsic stresses [44]. Factor Xa is the convergence point of the extrinsic and intrinsic components of the coagulation cascade. Several studies have shown that FXa can activate MAPKs. The MAPK and NF-κB pathways were found to be involved in FXa-induced TF expression in human umbilical endothelial cells [45]. In addition, the activation of MAPK by factor Xa leads to phosphorylation of the transcription factors that, in turn, leads to the expression of genes involved in DNA synthesis and cell proliferation [46]. FXa inhibitors may inhibit the MAPK pathway, which was previously demonstrated in the study by Hashikata et al. [47]. Thus, MAPK signaling pathways have been shown to be involved in the pathophysiology of thrombosis or platelet activity in a direct or indirect manner [48–53].

In healthy volunteers with 15 mg rivaroxaban, miR-483 levels were significantly higher in high- AXA_3h_ group, which also indicated the strong inhibition of FXa. Therefore, we speculated that the MAPK pathway was inhibited. We also found that miR-483 and miR-320a levels in the case group (a better response) measured 3 h after rivaroxaban administration was significantly higher than at baseline. MiR-483 levels also have the potential to differentiate AXA_3h_. We assume that higher miR-483 may lead to downregulation of its predicted target gene (MAPK3), thereby inhibiting the MAPK pathway, which may inhibit thrombosis directly or indirectly. Rivaroxaban, an FXa inhibitor, may also inhibit the MAPK pathway, resulting in an enhanced antithrombotic effect at the same time. The source of increased miRNA levels and their mechanisms correlated with PK/PD profiles requires further research.

3. The association of miR-320a and integrin subunit beta 3 (ITGB3)

Previous studies have validated that ITGB3 is the target gene of miR-320a [54, 55]. It was also in the top cluster in the MCODE analysis and a hub gene ranked by the CytoHubba plugin. Fager et al. found that platelets capable of binding FXa demonstrated significantly increased expression of key adhesion molecules, including integrin β3, thereby placing these platelets in a unique position to contribute to both hemostatic and thrombotic events [56]. Therefore, we assumed that the upward miR-320a level may down-regulate integrin β3 and may affect the adherent properties of platelets and their procoagulant response, which may lead to an enhanced antithrombotic effect of rivaroxaban.

Previous studies have reported that miR-320a and miR-483 are associated with atrial fibrillation or thrombotic disease. Circulating extracellular miR-320a-3p was elevated in paroxysmal AF patients compared to healthy controls and hypertensive patients without AF [57], and circulating miRNA-320a/b was differentially expressed in patients with deep vein thrombosis [58]. Elevated serum miR-483-5p levels may predict patients at risk for postoperative AF [20]. Since the level of miRNAs may also be regulated by disease status, the findings on healthy volunteers should be further validated in a larger patient cohort. The possible roles and mechanisms of these differentially expressed miRNAs should be further elucidated in patients taking rivaroxaban.

Above all, the goal of this study is to identify miRNA's potential as a biomarker for rivaroxaban therapeutic drug monitoring. The close correlation between miRNA and PK/PD parameters is the basis for this possibility. Differences in miRNA levels may influence the efficacy and safety after rivaroxaban therapy, which is particularly essential in critical situations, such as in patients with severe bleeding, for the detection of residual anticoagulant drug effects before surgery, prior to thrombolysis in acute stroke patients, after rescue from overdose, during the therapy in patients with extremes of body weight, to evaluate drug interactions, in cases of renal impairment, and in situations where there is a suspicion of non-compliance [59, 60]. Therefore, it is of great importance to discover novel biomarkers which could help distinguish subjects with different drug responses. According to our study, the likelihood that the effect of rivaroxaban is enhanced depending on the levels of miR-320a and miR-483 after administration. Given the potential link between AXA and bleeding incidents, it is also plausible that the higher miRNA levels may be associated with bleeding incidents. Therefore, in patients with higher miR-320a and miR-483 levels, particular attention should be paid to while using rivaroxaban. They may also become potential targets for rivaroxaban therapy to predict responses and help personalize pharmacotherapy.

Our study also had some limitations. First, although this study included healthy volunteers in three sub-centers, the number of subjects for microarray and RT-qPCR was still relatively small. miRNA levels in patients with and without bleeding were only compared in six matched pairs. The number of patients was too small to draw robust conclusions. Therefore, further evidence to verify the present findings is needed. Second, although miRNAs have the potential to predict several diseases, inconsistencies among detection devices and different procedures of data acquisition (such as various normalization methods for circulating miRNAs) hinder the wide use of miRNAs for clinical applications [61]. A previous study reported that repetitive freeze–thaw cycles may result in a significant reduction in miRNA concentration in both plasma and serum samples [62]. Thus, the methodological standards for quantifying miRNAs need to be further unified and improved so that the results can be interpreted accurately. Moreover, although pathway enrichment analysis has been frequently utilized to elucidate functional implications for dysregulated circulating miRNAs, this methodology cannot be free from inherent information bias and may provide inaccurate results [63]. The precise mechanism remains unknown. Further supporting data with experimental approaches will be necessary to verify the findings of our study.

## Conclusion

In conclusion, we found that miRNA impact the PK and PD profiles of rivaroxaban in healthy Chinese populations. MiR-320a and miR-483 may be novel biomarkers and potential targets for anticoagulation therapy involving rivaroxaban. Bioinformatic analysis showed that these miRNAs may play a regulatory role by targeting ABCG2, ITGB3, MAPK1/3, etc. However, no significant differences were found in the comparisons among patients. Further data are warranted to strengthen the robustness of the present findings and to analyze the role of miRNAs in predicting the PK or PD profiles and clinical outcomes of anticoagulants.

## Supplementary Information

**Additional file 1**. **Table S1**: The inclusion criteria of healthy volunteers and patients

**Additional file 2**. **Table S2**: Analytics for the assessment of rivaroxaban plasma concentrations in healthy volunteers in sub-centers

**Additional file 3**. **Table S3**: Comparison of plasma miR-320a and miR-483 levels between the case and control groups in healthy volunteers

**Additional file 4**. **Table S4**: Risk estimates of 3 h miRNA levels for rivaroxaban response by univariate logistic analysis in healthy volunteers

**Additional file 5**. **Table S5**: Plasma miR320a/miR-483 levels compared between different groups in patients

**Additional file 6**. **Table S6**: Risk estimates of 3 h miRNA levels for rivaroxaban response by univariate logistic analysis in patients

**Additional file 7**. **Table S7**: Comparison of plasma miR-320a and miR-483 levels between 3 and 0 h in healthy volunteers

**Additional file 8**. **Table S8**: Target analysis for miR-320a and miR-483

## Data Availability

All data generated or analysed during this study are included in this published article and its additional files.
