# Supplementary material for: Circulating miR-320a-3p and miR-483-5p level associated with pharmacokinetic–pharmacodynamic profiles of rivaroxaban
Source: Hum Genomics. 2022 Dec 28;16:72. doi: 10.1186/s40246-022-00445-5 (PMC9795792; doi:10.1186/s40246-022-00445-5)
Supplement: Supplementary file 1 — Additional file 1. Table S1: The inclusion criteria of healthy volunteers and patients [file 40246_2022_445_MOESM1_ESM.docx]

**Additional Table 1** The inclusion criteria of healthy volunteers and patients

For healthy volunteers, participants meeting the following inclusion criteria will be included in the study: (1) Subjects were required to be 18-45 years old and with a body mass index (BMI) ranging from 18 to 26 kg/m^2^. (2) The health status of participants was confirmed through medical history interview, physical examination, vital signs (blood pressure, pulse rate, and temperature), laboratory tests (blood chemistry, hematology, and urine analysis), and 12-lead electrocardiogram. (3) None of the subjects had been taking any medicine for at least 4 weeks prior to initiation of the study.

For patients, inclusion criteria were as follows: (1) rivaroxaban was taken for prevention of thrombosis in patients with nonvalvular atrial fibrillation (NVAF), prevention and treatment of deep vein thrombosis/pulmonary embolism and prevention of thrombosis after knee/hip replacement; (2) the patient’s age was > 18 years old, unlimited for gender. Baseline characteristics were recorded when patients were enrolled.
