# Supplementary material for: Circulating miR-320a-3p and miR-483-5p level associated with pharmacokinetic–pharmacodynamic profiles of rivaroxaban
Source: Hum Genomics. 2022 Dec 28;16:72. doi: 10.1186/s40246-022-00445-5 (PMC9795792; doi:10.1186/s40246-022-00445-5)
Supplement: Supplementary file 2 — Additional file 2. Table S2: Analytics for the assessment of rivaroxaban plasma concentrations in healthy volunteers in sub-centers [file 40246_2022_445_MOESM2_ESM.docx]

**Additional Table 2** Analytics for the assessment of rivaroxaban plasma concentrations in healthy volunteers in sub-centers

| **Drug** | **Center** | **Dose** | **LC/HPLC Conditions** | | |  | **MS** | |  | **Inter- and Intra-day Precision and Accuracy** | | |
| --- | --- | --- | --- | --- | --- | --- | --- | --- | --- | --- | --- | --- |
|  |  | **(mg)** | **Analytical Column** | **Mobile Phase A/B** | **Injection Volume, Flow Rate & Column Temperature** |  | **Quantitation & Scan Mode** | **Precursor- to - Production Pairs m/z** |  | **Linear Calibration Ranges**  **(ng/mL) ^*,†^** | **RSD** | **Accuracy** |
| Rivaroxaban | Center1 | 10 | ACQUITY UPLC® HSS T3 (1.8 µm, 2.1×50 mm) | 10 mM HCOONH4 in H2O/ CAN:H2O=9:1, v/v | 5.0 μL,  500 μL/min., 40˚C |  | AB SCIEX QTRAP 6500,  Positive scan mode | Rivaroxaban: 435.90/144.90  Internal standard: 440.00/144.90 |  | 0.500-400 | Inter-day <3.5%;  Intra-day <4.5% | Data not available |
| Rivaroxaban | Center2 | 15 | Shim-pack GIST，C8，（3 µm, 2.1×50 mm） | 0.1%FA in H2O/ 95%ACN | 10.0 μL,  400 μL/min., 45˚C |  | SHIMADZU LCMS-8060, Positive scan mode | Rivaroxaban: 436.00/144.95  Internal standard: 440.15/144.95 |  | 0.400-400 | <15% | 99.2-100% |
| Rivaroxaban | Center3 | 20 | Waters, Xbridge C18 (2.1X50mm), 3.5μm | 5 mM HCOONH4 in H2O/ 0.1% FA in ACN | 3.0 μL,  600 μL/min., 40˚C |  | API 5500 (AB Sciex)  Positive scan mode | Rivaroxaban: 436.1/145.0  Internal standard: 440.1/145.0 |  | 1.00-500 | Inter-day≤6.5%;  Intra-day ≤6.9% | 98.8-112.0% |

No significant matrix effect was found. All samples were analyzed within established storage stability periods.

HPLC, High-performance liquid chromatography; LC, Liquid chromatography; MS, Mass spectrometer.

^*^, QC sample concentrations of each sub-center: Center1: 1.50, 15.0 and 300 ng/mL; Center2: 0.400, 1.20, 150 and 300 ng/mL; Center3: 3.00, 25.0, 200 and 400 ng/mL.

^†^, Absolute recovery rates (mean±SD%) of each sub-center: Center1: 108.4 - 110.1% for 1.50, 15.0 and 300 ng/mL; Center2: 93.5±1.20%, 99.1±1.15% and 105.5±1.21% for 1.20, 150 and 300 ng/mL; Center3: 98.2%, 76.8% and 74.7%% for 3.00, 200 and 400 ng/mL.
