# Supplementary material for: Circulating miR-320a-3p and miR-483-5p level associated with pharmacokinetic–pharmacodynamic profiles of rivaroxaban
Source: Hum Genomics. 2022 Dec 28;16:72. doi: 10.1186/s40246-022-00445-5 (PMC9795792; doi:10.1186/s40246-022-00445-5)
Supplement: Supplementary file 4 — Additional file 4. Table S4: Risk estimates of 3 h miRNA levels for rivaroxaban response by univariate logistic analysis in healthy volunteers [file 40246_2022_445_MOESM4_ESM.docx]

**Additional Table 4** Risk estimates of 3 h miRNA levels for rivaroxaban response by univariate logistic analysis in healthy volunteers

| **miRNA** | **OR** | **95%CI** | **p value** |  | **OR** | **95%CI** | **p value** |
| --- | --- | --- | --- | --- | --- | --- | --- |
|  | **10mg: High AXA_3h_ vs Low AXA_3h_** | | |  | **10mg: High AUC_0-t_ vs Low AUC_0-t_** | | |
| **miR-320a ^a^** | 1.268 | 0.826-1.949 | 0.278 |  | 1.353 | 0.787-2.324 | 0.274 |
| **miR-483 ^a^** | 1.846 | 0.631-5.4 | 0.263 |  | 1.764 | 0.492-6.323 | 0.383 |
|  | **15mg: High AXA_3h_ vs Low AXA_3h_** | | |  | **15mg: High AUC_0-t_ vs Low AUC_0-t_** | | |
| **miR-320a ^a^** | 1.303 | 0.765-2.22 | 0.33 |  | 1.036 | 0.568-1.888 | 0.909 |
| **miR-483 ^a^** | 5.608 | 1.112-28.282 | **0.037^*^** |  | 1.503 | 0.761-2.969 | 0.241 |

^a^ MiRNAs were standardized (by interquartile range [IQR], respectively). Odds ratios are expressed per one IQR increment. OR: odds ratio; CI: confidence interval; AXA_3h_: anti-Xa activity measured 3h after rivaroxaban administration; AUC_0-t_: area under the plasma concentration time-curve from time 0 to time of last determinable concentration.
