# Supplementary material for: Circulating miR-320a-3p and miR-483-5p level associated with pharmacokinetic–pharmacodynamic profiles of rivaroxaban
Source: Hum Genomics. 2022 Dec 28;16:72. doi: 10.1186/s40246-022-00445-5 (PMC9795792; doi:10.1186/s40246-022-00445-5)
Supplement: Supplementary file 5 — Additional file 5. Table S5: Plasma miR320a/miR-483 levels compared between different groups in patients [file 40246_2022_445_MOESM5_ESM.docx]

| **Group** | **n** | **median** | **IQR** | **p value** |  | **median** | **IQR** | **p value** |
| --- | --- | --- | --- | --- | --- | --- | --- | --- |
|  |  | **miR-320a** | | |  | **miR-483** | | |
|  |  | **High AXA_3h_ vs Low AXA_3h_** | | | | | | |
| **Case** | 14 | 0.04188 | 0.09751 | 0.1139 |  | 0.002053 | 0.003411 | 0.7688 |
| **Control** | 14 | 0.1130 | 0.1384 |  |  | 0.002118 | 0.004738 |  |
|  |  | **Bleeding vs Non bleeding** | | | | | | |
| **Bleeding** | 6 | 0.09125 | 0.1164 | 0.3939 |  | 0.006072 | 0.008588 | 0.9372 |
| **Non-bleeding** | 6 | 0.04149 | 0.2535 |  |  | 0.004279 | 0.06737 |  |

**Additional Table 5** Plasma miR-320a/miR-483 levels compared between different groups in patients

IQR: interquartile range.
