# Supplementary material for: Circulating miR-320a-3p and miR-483-5p level associated with pharmacokinetic–pharmacodynamic profiles of rivaroxaban
Source: Hum Genomics. 2022 Dec 28;16:72. doi: 10.1186/s40246-022-00445-5 (PMC9795792; doi:10.1186/s40246-022-00445-5)
Supplement: Supplementary file 6 — Additional file 6. Table S6: Risk estimates of 3 h miRNA levels for rivaroxaban response by univariate logistic analysis in patients [file 40246_2022_445_MOESM6_ESM.docx]

**Additional Table 6** Risk estimates of 3 h miRNA levels for rivaroxaban response by univariate logistic analysis in patients

| **miRNA** | **OR** | **95% CI** | **p value** |  | **OR** | **95% CI** | **p value** |
| --- | --- | --- | --- | --- | --- | --- | --- |
|  | **High AXA_3h_ vs Low AXA_3h_** | | |  | **Bleeding vs Non bleeding** | | |
| **miR-320a ^a^** | 0.874 | 0.480-1.591 | 0.659 |  | 0.820 | 0.413-1.628 | 0.571 |
| **miR-483 ^a^** | 0.938 | 0.786-1.119 | 0.476 |  | 0.590 | 0.224-1.552 | 0.285 |

^a^ MiRNAs were standardized (by interquartile range [IQR], respectively). Odds ratios are expressed per one IQR increment. OR: odds ratio; CI: confidence interval; AXA: anti-Xa activity.
