# Supplementary material for: Circulating miR-320a-3p and miR-483-5p level associated with pharmacokinetic–pharmacodynamic profiles of rivaroxaban
Source: Hum Genomics. 2022 Dec 28;16:72. doi: 10.1186/s40246-022-00445-5 (PMC9795792; doi:10.1186/s40246-022-00445-5)
Supplement: Supplementary file 7 — Additional file 7. Table S7: Comparison of plasma miR-320a and miR-483 levels between 3 and 0 h in healthy volunteers [file 40246_2022_445_MOESM7_ESM.docx]

**Additional Table 7** Comparison of plasma miR-320a and miR-483 levels between 3 h and 0 h in healthy volunteers

| **Group** | **n** | **median** | **IQR** | **p value** |  | **Group** | **n** | **median** | **IQR** | **p value** |  | **Group** | **n** | **median** | **IQR** | **p value** |  | **Group** | **n** | **median** | **IQR** | **p value** |
| --- | --- | --- | --- | --- | --- | --- | --- | --- | --- | --- | --- | --- | --- | --- | --- | --- | --- | --- | --- | --- | --- | --- |
| **miR-320a** | | | | | | | | | | |  | **miR-483** | | | | | | | | | | |
| **10mg** | | | | | | | | | | |  | **10mg** | | | | | | | | | | |
| **3h vs 0h: Group classified by AXA_3h_** | | | | |  | **3h vs 0h: Group classified by AUC_0-t_** | | | | |  | **3h vs 0h: Group classified by AXA_3h_** | | | | |  | **3h vs 0h: Group classified by AUC_0-t_** | | | | |
| **Fasted** | | | | |  | **Fasted** | | | | |  | **Fasted** | | | | |  | **Fasted** | | | | |
| case-3h | 5 | 0.1548 | 0.1283 | 0.6250 |  | case-3h | 5 | 0.0859 | 0.1492 | 0.4375 |  | case-3h | 5 | 0.0029 | 0.0066 | 0.4375 |  | case-3h | 5 | 0.0011 | 0.0049 | 0.6250 |
| case-0h | 5 | 0.1667 | 0.3979 |  |  | case-0h | 5 | 0.1526 | 0.3782 |  |  | case-0h | 5 | 0.0018 | 0.0027 |  |  | case-0h | 5 | 0.0021 | 0.0025 |  |
| control-3h | 5 | 0.1749 | 0.5364 | 0.1250 |  | control-3h | 5 | 0.3229 | 0.5584 | 0.1250 |  | control-3h | 5 | 0.0047 | 0.0079 | >0.9999 |  | control-3h | 5 | 0.0042 | 0.0082 | 0.6250 |
| control-0h | 5 | 0.0432 | 0.0256 |  |  | control-0h | 5 | 0.0432 | 0.0339 |  |  | control-0h | 5 | 0.0064 | 0.0089 |  |  | control-0h | 5 | 0.0042 | 0.0053 |  |
| **Fed** | | | | |  | **Fed** | | | | |  | **Fed** | | | | |  | **Fed** | | | | |
| case-3h | 5 | 2.0130 | 9.5259 | 0.1875 |  | case-3h | 5 | 2.0130 | 9.0961 | 0.0625 |  | case-3h | 5 | 0.0353 | 0.2067 | 0.1875 |  | case-3h | 5 | 0.0109 | 0.1950 | 0.0625 |
| case-0h | 5 | 0.0961 | 0.0830 |  |  | case-0h | 5 | 0.0829 | 0.0387 |  |  | case-0h | 5 | 0.0012 | 0.0066 |  |  | case-0h | 5 | 0.0005 | 0.0019 |  |
| control-3h | 5 | 0.0937 | 0.1435 | 0.6250 |  | control-3h | 5 | 0.0848 | 0.1309 | 0.8125 |  | control-3h | 5 | 0.0003 | 0.0005 | 0.0625 |  | control-3h | 5 | 0.0003 | 0.0005 | 0.0625 |
| control-0h | 5 | 0.1241 | 0.0783 |  |  | control-0h | 5 | 0.1241 | 0.0894 |  |  | control-0h | 5 | 0.0006 | 0.0029 |  |  | control-0h | 5 | 0.0026 | 0.0029 |  |
| **Total** | | | | |  | **Total** | | | | |  | **Total** | | | | |  | **Total** | | | | |
| case-3h | 10 | 0.1610 | 2.1317 | 0.4316 |  | case-3h | 10 | 0.2081 | 1.9733 | 0.1934 |  | case-3h | 10 | 0.0045 | 0.0396 | 0.1309 |  | case-3h | 10 | 0.0025 | 0.0161 | 0.1934 |
| case-0h | 10 | 0.1048 | 0.1264 |  |  | case-0h | 10 | 0.0872 | 0.0860 |  |  | case-0h | 10 | 0.0015 | 0.0028 |  |  | case-0h | 10 | 0.0014 | 0.0021 |  |
| control-3h | 10 | 0.1189 | 0.2185 | 0.0645 |  | control-3h | 10 | 0.0991 | 0.2673 | 0.1055 |  | control-3h | 10 | 0.0018 | 0.0056 | 0.4922 |  | control-3h | 10 | 0.0018 | 0.0053 | 0.5566 |
| control-0h | 10 | 0.0614 | 0.0882 |  |  | control-0h | 10 | 0.0603 | 0.0882 |  |  | control-0h | 10 | 0.0028 | 0.0061 |  |  | control-0h | 10 | 0.0028 | 0.0042 |  |
| **15mg** | | | | | | | | | | |  | **15mg** | | | | | | | | | | |
| **3h vs 0h: Group classified by AXA_3h_** | | | | |  | **3h vs 0h: Group classified by AUC_0-t_** | | | | |  | **3h vs 0h: Group classified by AXA_3h_** | | | | |  | **3h vs 0h: Group classified by AUC_0-t_** | | | | |
| **Fasted** | | | | |  | **Fasted** | | | | |  | **Fasted** | | | | |  | **Fasted** | | | | |
| case-3h | 7 | 0.1236 | 0.0949 | **0.0156^*^** |  | case-3h | 7 | 0.0693 | 0.1240 | **0.0156^*^** |  | case-3h | 7 | 0.0060 | 0.0065 | **0.0156^*^** |  | case-3h | 7 | 0.0040 | 0.0058 | **0.0313^*^** |
| case-0h | 7 | 0.0223 | 0.0133 |  |  | case-0h | 7 | 0.0210 | 0.0150 |  |  | case-0h | 7 | 0.0013 | 0.0015 |  |  | case-0h | 7 | 0.0013 | 0.0009 |  |
| control-3h | 7 | 0.0388 | 0.2809 | 0.2969 |  | control-3h | 7 | 0.0233 | 0.2878 | >0.9999 |  | control-3h | 7 | 0.0016 | 0.0021 | 0.6875 |  | control-3h | 7 | 0.0013 | 0.0018 | 0.3750 |
| control-0h | 7 | 0.0263 | 0.0663 |  |  | control-0h | 7 | 0.0328 | 0.0593 |  |  | control-0h | 7 | 0.0019 | 0.0025 |  |  | control-0h | 7 | 0.0019 | 0.0046 |  |
| **Fed** | | | | |  | **Fed** | | | | |  | **Fed** | | | | |  | **Fed** | | | | |
| case-3h | 7 | 0.1511 | 0.8987 | 0.2188 |  | case-3h | 7 | 0.3154 | 0.6269 | **0.0156^*^** |  | case-3h | 7 | 0.0065 | 0.0073 | >0.9999 |  | case-3h | 7 | 0.0077 | 0.0133 | 0.0781 |
| case-0h | 7 | 0.0524 | 0.0357 |  |  | case-0h | 7 | 0.0280 | 0.0162 |  |  | case-0h | 7 | 0.0024 | 0.0072 |  |  | case-0h | 7 | 0.0024 | 0.0026 |  |
| control-3h | 7 | 0.0308 | 0.0182 | 0.8125 |  | control-3h | 7 | 0.0308 | 0.4233 | 0.4688 |  | control-3h | 7 | 0.0020 | 0.0026 | 0.9375 |  | control-3h | 7 | 0.0018 | 0.0008 | 0.3750 |
| control-0h | 7 | 0.0334 | 0.1322 |  |  | control-0h | 7 | 0.0391 | 0.0185 |  |  | control-0h | 7 | 0.0023 | 0.0034 |  |  | control-0h | 7 | 0.0027 | 0.0038 |  |
| **Total** | | | | |  | **Total** | | | | |  | **Total** | | | | |  | **Total** | | | | |
| case-3h | 14 | 0.1322 | 0.2258 | **0.0085^*^** |  | case-3h | 14 | 0.1050 | 0.3026 | **0.0001^***^** |  | case-3h | 14 | 0.0063 | 0.0064 | 0.0906 |  | case-3h | 14 | 0.0063 | 0.0087 | **0.0017^**^** |
| case-0h | 14 | 0.0347 | 0.0435 |  |  | case-0h | 14 | 0.0256 | 0.0169 |  |  | case-0h | 14 | 0.0020 | 0.0022 |  |  | case-0h | 14 | 0.0017 | 0.0017 |  |
| control-3h | 14 | 0.0333 | 0.1149 | >0.9999 |  | control-3h | 14 | 0.0290 | 0.3218 | 0.6257 |  | control-3h | 14 | 0.0018 | 0.0020 | 0.7148 |  | control-3h | 14 | 0.0015 | 0.0013 | 0.2412 |
| control-0h | 14 | 0.0298 | 0.0818 |  |  | control-0h | 14 | 0.0360 | 0.0316 |  |  | control-0h | 14 | 0.0021 | 0.0027 |  |  | control-0h | 14 | 0.0025 | 0.0040 |  |

IQR: interquartile range; AXA_3h_: anti-Xa activity measured 3h after rivaroxaban administration; AUC_0-t_: area under the plasma concentration-time curve from time 0-t h; ^*^: p<0.05; ^**^: p<0.01; ^***^: p<0.001
