# Supplementary material for: Circulating miR-320a-3p and miR-483-5p level associated with pharmacokinetic–pharmacodynamic profiles of rivaroxaban
Source: Hum Genomics. 2022 Dec 28;16:72. doi: 10.1186/s40246-022-00445-5 (PMC9795792; doi:10.1186/s40246-022-00445-5)
Supplement: Supplementary file 8 — Additional file 8. Table S8: Target analysis for miR-320a and miR-483 [file 40246_2022_445_MOESM8_ESM.docx]

**Additional Table 8** Target analysis for miR-320a and miR-483

| **miRNA name** | **Number of target genes predicted in any algorithm ^a^** | **Genes predicted in all three algorithms ^a^** | **Validated target genes ^b^** |
| --- | --- | --- | --- |
| hsa-miR-320a-3p | 2409 | 122: ADAM10, AK4, AKT3, ANKFY1, ANKRD52, ARL5A, ARL8B, ARPP19, ATRX, AZIN1, BANP, BMI1, CISD2, CKAP5, COPS2, CPD, CREB3L2, CREBRF, CRKL, CYLD, DCBLD2, DLX1, DNAJA2, DNAJC3, DR1, EIF3J, ELOVL6, ENAH, ENPEP, EYA3, FAM117B, FBXO28, FOXM1, GNAI1, GNS, GOLGA1, GPBP1, GTPBP2, GXYLT1, HECTD2, HIVEP2, HLTF, HNRNPF, HOXA10, HOXB4, HSPA4, HSPH1, IGF2BP3, INO80D, IPO5, KCNAB1, KITLG, KLHL14, KLHL15, KRI1, LPIN3, MAK16, MAPK1, MAPK8IP3, MIER1, MITF, MMP16, MRFAP1, MTDH, MTMR12, MXI1, NAA40, NCAPD3, NFIA, NRP1, NXT2, PAN3, PBX3, PCGF5, PCSK1, PDCD4, PFKM, PHC1, PI15, PIK3R1, PLXNC1, POLR1C, PSMF1, PTEN, RAB11A, RANBP2, RASA1, RNF185, RPL15, SATB2, SEMA3A, SERINC3, SESTD1, SF3B3, SLC5A3, SLC6A8, SOX4, SREK1, SRP19, ST3GAL1, STAG2, STARD4, SYNGR2, TANC1, TANC2, TMEM106B, TMEM64, TOR1AIP2, TPD52, TRIAP1, TSC1, ULK1, VDAC1, VPS36, WDR1, WWC2, XPO1, YIPF6, YOD1, YWHAH, ZDHHC3, ZNF652 | 30: AQP1, AQP4, ARF1, BANP, BMI1, FOXM1, GNAI1, HSPB6, IGF1R, ITGB3, KITLG, MAPK1, MCL1, MTDH, NFATC3, NPR1, NRP1, PBX3, PDCD4, POLR3D, PTEN, RAB14, RAC1, RUNX2, SUZ12, TAC1, TFRC, TRPC5, VDAC1, YWHAZ |
| hsa-miR-483-5p | 1861 | 19: ALCAM, CBS, CUTA, DLG5, FAM160B2, FN3K, HGSNAT, HR, MAP4K4, MAPK3, MSC, RUSC1, SMG6, STK40, STX6, TIMP2, TMCC2, WDR92, ZMYM6 | 4: MAPK3, SRF, FAM160B2, ALCAM |

^a^Targetscan version 8.0 (http://www.targetscan.org/vert_80/), miRTarBase version 8.0 (http://miRTarBase.cuhk.edu.cn/), and miRDB, version 6.0 (<http://mirdb.org>).

^b^Validated miRNA-target interactions with western blot, qPCR, and/or reporter assay were shown in miRTarBase 8.0.
